# The Effect of Steroid Treatment on Lipocortin Immunoreactivity of Rat Brain

**DOI:** 10.1155/S0962935194000232

**Published:** 1994

**Authors:** K. G. Go, J. G. Ter Haar, L. de Ley, F. Zuiderveen, L. Parente, E. Solito, W. M. Molenaar

**Affiliations:** 1 Department of Neurosurgery University of Groningen The Netherlands; 2 Department of Clinical Immunology University of Groningen The Netherlands; 3 Department of Pathology University of Groningen The Netherlands; 4 lmmunobiological Research Institute Siena Italy

## Abstract

Lipocortin-1, lipocortin-2 and lipocortin-5 were immunohistochemically assessed in rats. Apart from animals receiving no treatment, other animals received pretreatment with methylprednisolone, or the 21-aminosteroid U-74389F. Whereas Hpocortin immunoreactivity was absent in the greater part of the brain in animals not pretreated with steroid (except in sporadic microglial cells and choroid plexus), there was obvious immunostaining of parenchymatous elements in steroid pretreated animals. In the steroid pretreated animals lipocortin immunoreactivity of the brain tissue may indicate local formation of lipocortin under the influence of steroids that had entered the tissue. The cellular elements which showed immunostaining included meningeal cells, neurones, ependyma, oligodendroglia and capillary endotheHum.


Mediators of Inflammation 3, 177–180 (1994)

**Lipocortin-1, lipocortin-2 and lipocortin-5 were immunohistochemically assessed in rats. Apart from animals receiving no treatment, other animals received pretreatment with methylprednisolone, or the 21-aminosteroid U-74389F. Whereas lipocortin immunoreactivity was absent in the greater part of the brain in animals not pretreated with steroid (except in sporadic microglial cells and choroid plexus), there was obvious immunostaining of parenchymatous elements in steroid pretreated animals. In the steroid pretreated animals lipocortin immunoreactivity of the brain tissue may indicate local formation of lipocortin under the influence of steroids that had entered the tissue. The cellular elements which showed immunostaining included meningeal cells, neurones, ependyma, oligodendroglia and capillary endothelium.**

**Key words:** 21-Aminosteroids, Brain oedema, Glucocorticosteroids, Lipocortins in brain

### The effect of steroid treatment on lipocortin immunoreactivity of rat brain

K. G. Go,[1,CA] J. G. Ter Haar,[2] L. de Ley,[2] F. Zuiderveen,[1] L. Parente,[4] E. Solito[4] and W. M. Molenaar[3]

Departments of [1]Neurosurgery, [2]Clinical Immunology, and [3]Pathology, University of Groningen, The Netherlands; [4]Immunobiological Research Institute Siena, Italy

[CA] Corresponding Author

### Introduction

Glucocorticosteroids have been shown clinically to exert a dramatic effect on certain types of brain oedema of vasogenic origin, i.e. brain oedema that has been caused by leakage of blood plasma into brain tissue due to breakdown of the blood–brain barrier.[1] In this respect, vasogenic brain oedema resembles the inflammatory exudate in other parts of the body, which in inflammatory lesions results from the increase of vascular permeability. Blood–brain barrier disruption often accompanies brain tumours, traumatic lacerations, haemorrhages and infarctions, and therefore brain oedema has a considerable clinical significance, especially in view of the restricted expansive capacity of the head, contributing to morbidity and eventual mortality.[2,3] It has been shown that the effect of glucocorticosteroids upon inflammation is based upon the formation of lipocortins (LCT), proteins that inhibit the enzyme phospholipase $A_2$.[4,5] This enzyme is supposed to play a role in inflammatory processes by liberating arachidonic acid from membrane phospholipids, while arachidonic acid is known to serve as a precursor for eicosanoids (prostaglandins, leukotrienes and lipoxins) which are considered as mediators of inflammation. Arachidonic acid has been measured in increased amounts in brain tissue that has been injured by various means, while arachidonic acid itself has been shown to be capable of inducing brain oedema, both *in vitro* and *in vivo* following its intracerebral administration.[6–8]

Clinically, glucocorticosteroids have been shown to exert a most beneficial influence in the clinical treatment of tumour-associated oedema, where it is effective in low dose. In contrast, they have been shown to be ineffective in reducing mortality in brain trauma, and to be of doubtful efficacy in stroke.[3] Currently, the group of 21-aminosteroid compounds has been introduced that is supposed to exert a similar protective effect on traumatized neural tissue while lacking the other glucocorticoid effects.[9]

Lipocortins (also called annexins) constitute a family of proteins, the members of which have been identified by analysis of the sequences. Lipocortins 1–5 (LCT 1–5) have in common a core with four repeats of 70 amino acids, whereas the core of LCT-6 contains eight repeats. It is the N-terminus that is variable, being longest in LCT-1 and decreasing in length in the order of LCT-2, LCT-6, LCT-3, LCT-5 and LCT-4.[10] Since the LCTs are polypeptides of 32–67 kDa they probably cannot cross the intact blood–brain barrier. If they are to be assigned a role in mediating the effects of glucocorticoids in brain tissue, that should imply their local formation in the blood–brain barrier or in the brain parenchyma.

### Materials and Methods

The studies were performed on adult Wistar rats of 180 g mean body weight.

*Steroids and dosage:* Apart from untreated animals, the animals consisted of a group treated with 2 mg/kg of methylprednisolone, and a 21-aminosteroid (U-74389F, Upjohn Co.; 10 mg/kg), the day before and 2 h before killing. The animals were killed by decapitation, and the ipsilateral cerebral hemisphere was

© 1994 Rapid Communications of Oxford Ltd quickly frozen in Freon. Frozen sections of 5 μm thickness were cut and treated with anti-lipocortin antibody at 1:20 dilution.

*Binding of antibody:* Binding of antibody was visualized by horseradish peroxidase-conjugated swine anti-rabbit antibody (Dako, 1:40) developed with 2-aminoethyl-carbazole + $H_2O_2$. The sections were weakly counterstained with haematoxylin.[11] As controls the samples were incubated with phosphate buffered saline (PBS) as the first step.

*Anti-lipocortin antibodies:* The anti-lipocortin antibodies were polyclonal antibodies that had been raised in rabbits against either recombinant human LCT-2, or the amino terminus peptides of LCT-1 and LCT-5 (amino acids 15–31 for LCT-1, and amino acids 1–11 for LCT-5). For immunization the peptides (1 mg/ml) were crosslinked to 400 μg keyhole limpet haemocyanin by incubation with 0.5 ml of 20 mM glutaraldehyde for 30 min at room temperature. In Western blotting experiments the antibodies recognized the different proteins with no cross reactivity.

### Results

In the untreated animals LCT-1 immunoreactivity could not be observed in the greater part of the brain, but only in sporadic cells, which are probably of microglial nature (Fig. 1). The choroid plexus showed a faint reaction (not shown). LCT-2 immunostaining was similar although less pronounced.

The rats pretreated with methylprednisolone showed widespread LCT-1 immunoreactivity in neurones, especially in those of the hippocampus, and probably also in astrocytes as well as in microglial cells (Fig. 2). Also, the choroid plexus showed LCT-1 immunoreactivity.

Oligodendrocytes as well as capillary endothelial cells were devoid of reaction product. LCT-2 immunostaining was less pronounced in the nervous parenchyma, but was well demonstrable in the choroid plexus and the leptomeninges (Fig. 3). LCT-2 immunoreactivity was far less pronounced than the reactivity to LCT-1, with the strongest reaction seen in the meninges. The immunoreactivity to LCT-5 was similar to that of LCT-1, being exhibited most by microglia, the leptomeninges and also by some blood vessels.

Pretreatment with the 21-aminosteroid gave the strongest LCT-1 immunoreactivity in cortical neurones, oligodendrogial cells of the white matter (Fig. 4), ventricular ependyma (Fig. 5) and choroid plexus (Fig. 6). After the 21-aminosteroid pretreatment LCT-2 immunoreactivity was evident also in cortical neurones (Fig. 7), capillary endothelium, meningeal cells and ventricular ependyma. The controls incubated with PBS were all negative.

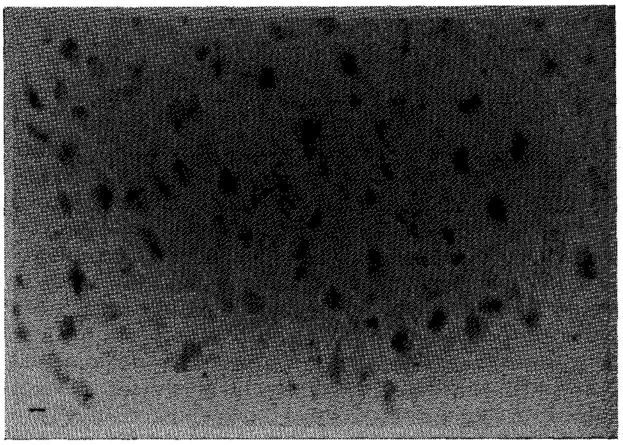

FIG. 1. Absence of LCT-1 immunostaining in brain parenchyma, except of sporadic microglia (arrows) in untreated uninjured animal. Bar, 10 μm.

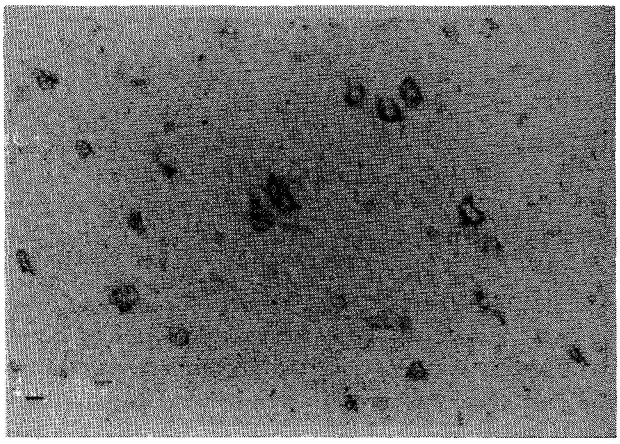

FIG. 2. LCT-1 immunostaining of neurones in normal cerebral cortex of rat treated with low-dose methylprednisolone. Bar, 10 μm.

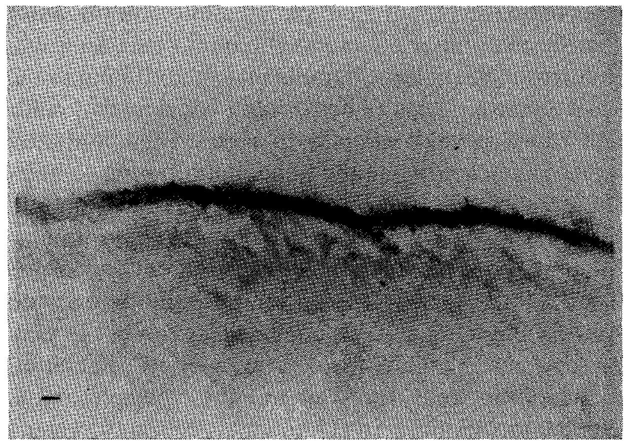

FIG. 3. LCT-2 immunostaining of leptomeninges in normal cerebral cortex of rat treated with low-dose methylprednisolone. Bar, 10 μm.

### Discussion

There is strong enhancement of LTC immunoreactivity in the nervous parenchyma of the glucocorticosteroid and 21-aminosteroid pretreated animals in contrast to the absence of spontaneous immunoreactivity in the majority of the brain of

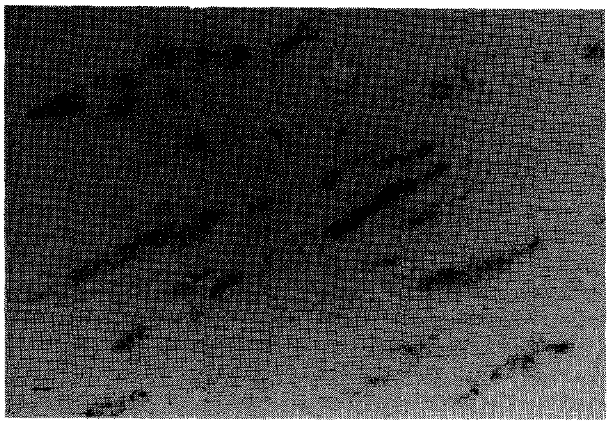

FIG. 4. LCT-1 immunoreactivity of rows of oligodendroglial cells in subcortical white matter of rat treated with 21-aminosteroid. Bar, 10 μm.

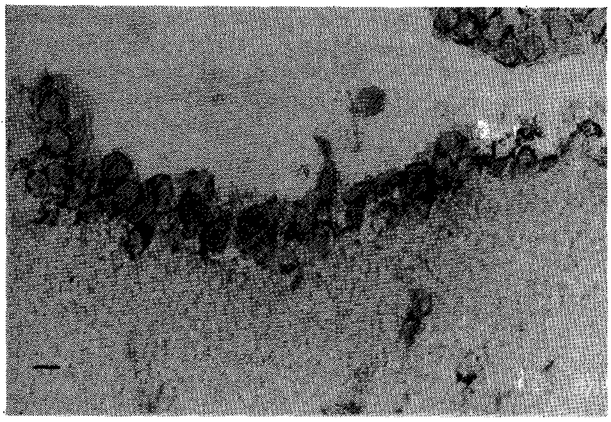

FIG. 5. LCT-1 immunostaining of ventricular ependyma of rat treated with 21-aminosteroid. Bar, 10 μm.

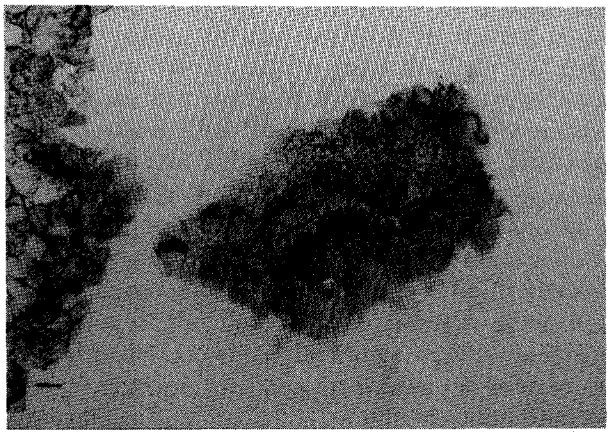

FIG. 6. LCT-1 immunostaining of choroid plexus of rat treated with 21-aminosteroid. Bar, 10 μm.

untreated animals. Whereas in untreated animals immunoreactivity was only localized in sporadic microglial cells, the enhanced immunoreactivity in steroid pretreated animals resided in neurones and probably astrocytes as well, and in oligodendrocytes in the 21-aminosteroid pretreated animals. The choroid plexus consistently showed immunoreactivity, even in animals not treated with steroid, as has been reported previously.[12] As the choroid plexus is one of the circumventricular organs possessing capillaries devoid of barrier properties, there is a possibility that the immunostaining may reflect LCT of systemic origin. Immunostaining of ventricular ependymal cells as reported previously[12,13] was seen after 21-aminosteroid pretreatment. Immunoreactivity to LCT-2 in capillary endothelium was only seen after 21-aminosteroid pretreatment.

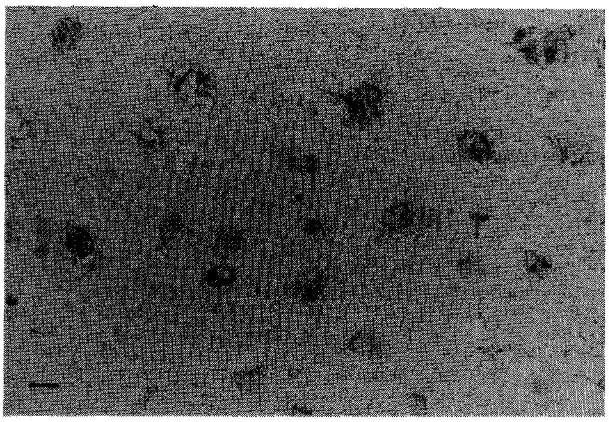

FIG. 7. LCT-2 immunostaining of cortical neurones of rat treated with 21-aminosteroid. Bar, 10 μm.

The results after steroid pretreatment clearly indicate the ability of the steroids to cross the blood–brain barrier, which was to be expected in view of their lipophilicity. From the order of appearance of immunostaining, it seems that the following order of inducibility of LCT formation may be derived: microglia, choroid plexus epithelium, meningeal cells, neurone/astrocyte, ependyma, oligodendroglia, capillary endothelium. The preferential effect of the steroids on microglia is well in accordance with previous findings of a preferential effect on macrophages.[14] The immunoreactivity was strongest for LCT-1 and less pronounced for LCT-2 and LCT-5.

Although the 21-aminosteroids have been reported to exert no action upon glucose metabolism, they had a strong effect in inducing LCT formation. In the present study the steroids were administered 1 day and 2 h prior to the experiment. Where dexamethasone had been administered to the animals only 2 h before termination, the pattern of LCT immunoreactivity was not different from that in untreated animals in that it was only observed in certain regions, being absent in the larger part of the brain.[13]

Although lately the effect of glucocorticosteroids in suppressing eicosanoid formation has been questioned in a number of cell types, the effect has been confirmed in macrophages,[14,15] to which the microglia of the central nervous system may be considered similar. It may be concluded that there is little spontaneous LCT immunoreactivity in the brain parenchyma, and that glucocortico- and 21-aminosteroids can induce LCT formation in the brain parenchyma, and may in this way mediate the effects of steroids in brain oedema.

### References

1. Klatzo I. Neuropathological aspects of brain edema. *J Neuropath Exp Neurol* 1967; **26**: 1–14.
2. Go KG. Pathophysiological aspects of brain edema. *Clin Neurol Neurosurg* 1984; **86**: 77–80.
3. Go KG. *Cerebral Pathophysiology. An integral approach with some emphasis upon clinical implications*. Amsterdam: Elsevier, 1992, 1–432.
4. Blackwell GJ, Carnuccio R, Di Rosa M, Flower RJ, Parente L, Persico P. Macrocortin: a polypeptide causing the anti-phospholipase effect of glucocorticoids. *Nature* 1980; **287**: 147–149.
5. Parente L, Becherucci C, Perretti M, *et al.* Are the lipocortins the second messengers of the anti-inflammatory action of glucocorticoids? In: Melli M, Parente L (Eds.) *Cytokines and Lipocortins in Inflammation and Differentiation*. New York: Wiley-Liss, 1990; 55–68.
6. Chan PH, Fishman RA. Brain edema: induction in cortical slices by polyunsaturated fatty acids. *Science* 1978; **201**: 358–360.
7. Chan PH, Fishman RA, Caronna J, Schmidley JW, Prioleau G, Lee J. Induction of brain edema following intracerebral injection of arachidonic acid. *Ann Neurol* 1983; **13**: 625–632.
8. Chan PH, Longar S, Fishman RA. Phospholipid degradation and edema development in cold-injured rat brain. *Brain Research* 1983; **277**: 329–337.
9. Hall ED, McCall JM, Yonkers PA, Chase RL, Braughler JM. A nonglucocorticoid analog of methylprednisolone duplicates its high dose pharmacology in models of CNS trauma and neuronal membrane damage. *J Pharmacol Exp Ther* 1987; **242**: 137–142.
10. Pepinsky RB, Tizard R, Mattaliano RJ, *et al.* Five distinct calcium and phospholipid binding proteins share homology with lipocortin I. *J Biol Chem* 1988; **263**: 10799.
11. De Ley L, Poppema S, Klein Nulend J, ter Haar JG, Schwander E, The TH. Immunoperoxidase staining on frozen tissue sections as a first screening assay in the preparation of monoclonal antibodies against small cell carcinoma of the lung. *Eur J Clin Oncol* 1984; **20**: 123–128.
12. Johnson MD, Kamso-Pratt JM, Whetsell WO, Pepinsky RB. Lipocortin-1 immunoreactivity in the normal human central nervous system and lesions with astrocytes. *Am J Clin Pathol* 1989; **92**: 424–429.
13. Strijbos PJLM, Tilders FJH, Carey F, Forder R, Rothwell NJ. Localization of immunoreactive lipocortin-1 in the brain and pituitary gland of the rat. Effects of adrenalectomy, dexamethasone and colchicine treatment. *Brain Research* 1991; **553**: 249–260.
14. Solito E, Raugei G, Melli M, Parente L. Dexamethasone induces the expression of the mRNA of lipocortin 1 and 2 and the release of lipocortin 1 and 5 in differentiated, but not undifferentiated U-937 cells. *FEBS Lett* 1991; **291**: 238–244.
15. Sebaldt RJ, Sheller JR, Oates JA, Roberts LJ, Fitzgerald GA. Inhibition of eicosanoid biosynthesis by glucocorticoids in humans. *Proc Natl Acad Sci USA* 1990; **87**: 6975–6978.

**Received 5 January 1994; accepted 25 January 1994**
